# Strain-specific and pooled genome sequences for populations of *Drosophila melanogaster* from three continents.

**DOI:** 10.12688/f1000research.6090.1

**Published:** 2015-01-29

**Authors:** Casey M. Bergman, Penelope R. Haddrill

**Affiliations:** 1 Faculty of Life Sciences, University of Manchester, Manchester, M13 9PT, UK; 2 Institute of Evolutionary Biology, School of Biological Sciences, University of Edinburgh, Edinburgh, EH9 3FL, UK

**Keywords:** Drosophila melanogaster, Wolbachia pipientis, population genomics, population genetics, pool-seq, DNA-seq

## Abstract

To contribute to our general understanding of the evolutionary forces that shape variation in genome sequences in nature, we have sequenced genomes from 50 isofemale lines and six pooled samples from populations of *Drosophila melanogaster* on three continents. Analysis of raw and reference-mapped reads indicates the quality of these genomic sequence data is very high. Comparison of the predicted and experimentally-determined *Wolbachia* infection status of these samples suggests that strain or sample swaps are unlikely to have occurred in the generation of these data. Genome sequences are freely available in the European Nucleotide Archive under accession ERP009059. Isofemale lines can be obtained from the *Drosophila* Species Stock Center.

## Introduction

Whole genome shotgun sequences can now be generated easily using short-read sequencing technology for most organisms. Hundreds of resequenced genomes now exist for *Drosophila melanogaster* that can be used for population and genomic analysis in this model insect species ( Lack *et al.*, 2014). To contribute to the worldwide sampling of population genomic data in *D. melanogaster*, we have sequenced genomes of multiple isofemale lines from three populations collected on different continents reported in Verspoor & Haddrill (2011): Montpellier, France (FR, n=20), Athens, Georgia, USA (GA, n=15) and Accra, Ghana (GH, n=15). Pools of these same isofemale lines were also sequenced to be able compare results based on strain-specific sequencing to pooled sequencing. Strains sequenced here were chosen because isofemale lines exist in the *Drosophila* Species Stock Center and because their infection status for the *Wolbachia pipientis* bacterial endosymbiont had previously been determined ( Verspoor & Haddrill, 2011).

## Materials and methods

Isofemale strains were selected randomly from the full population samples reported in Verspoor & Haddrill (2011). Genomic DNA for isofemale lines was prepared by snap freezing females in liquid nitrogen, then extracting DNA using a standard phenol-chloroform extraction protocol with ethanol and ammonium acetate precipitation. DNA samples were generated for each isofemale lines using 50, 25, and 25 adult females for the FR, GA and GH populations, respectively.

For pooled samples, single adult females from each isofemale line were used to construct two samples for each population. The first pooled sample contains one fly from each of the same strains that were sequenced as isofemale lines (FR_pool_20, GA_pool_15, GH_pool_15). The second pooled sample contains one fly from all isofemale lines sampled for each population reported in Verspoor & Haddrill (2011) (FR_pool_39, GA_pool_30, GH_pool_32).

500 bp short-insert libraries using the Illumina Paired-End Sample Prep Kit (Part # 1005063) were constructed and 90 bp paired-end reads were generated using an Illumina HiSeq 2000 to an estimated coverage of ~50× per strain by BGI-Hong Kong. Forty-one samples were sequenced in single lanes shared typically with two other samples on a single run and 15 samples were sequenced using the same layout on two runs, generating 71 pairs of fastq files for the 56 samples. Data were generated over a total of seven sequencing runs. Raw data was filtered by BGI to remove read pairs where either read contained adapters or greater than 50% of bases with a quality value <= 5. No other trimming or filtering of the raw data was performed prior to submission using original filenames provided by BGI to the European Nucleotide Archive.

## Dataset validation

To validate the quality of the raw sequence data, forward and reverse reads were analyzed using fastQC (version 0.11.2) ( http://www.bioinformatics.babraham.ac.uk/projects/fastqc/). Forward and reverse read files for all runs had PASS status for most fastQC statistics. Per base sequence quality gave FAIL status for forward or reverse read files for all of the GA samples (which were sequenced together on one run) because of poor quality scores in the terminal 1–5 bp of the read. These poor quality termini can be easily trimmed and do not affect mappability, as the percent of reads mapped for these runs is very high (see Dataset 1).

Descriptive statistics for validation of *Drosophila melanogaster* genome sequence data The PercentMapped column is obtained from the output of samtools flagstat using BAM files of mapped reads generated by bowtie2. The WolbachiaDepth, WolbachiaBreadth and PredictedInfectionStatus columns are obtained from the output of bedtools genomecov using BAM files of mapped reads generated by bowtie2. The ExperimentalInfectionStatus column is obtained from the results of Verspoor & Haddrill (2011). All other columns are obtained from the output of fastQC on the raw, unmapped reads.

To validate that the majority of the DNA sequenced is from the focal organism(s), untrimmed reads for each sample were mapped in paired-end mode using Bowtie (version 2.2.4) ( Langmead & Salzberg, 2012) with default options to a “hologenome” reference generated by concatenating genome sequences for *D. melanogaster* (Genbank accession GCA_000001215.4) ( Hoskins *et al.*, 2015) and *W. pipientis* (Genbank accession AE017196) ( Wu *et al.*, 2004). Mapping to a hologenome was performed since many of these strains are known to be infected with *Wolbachia* ( Verspoor & Haddrill, 2011). Unfiltered BAM files were used to estimate the proportion of reads in each sample that mapped to the expected target organisms using samtools flastat (version 0.1.19-44428cd) ( Li *et al.*, 2009). Greater than 96.8% of all reads in each run were mapped to the hologenome reference, indicating low levels of contaminating DNA in these data ( Dataset 1).

Mapping to a hologenome also allowed us to verify if strain or sample swaps occurred in the process of producing these genome sequences by comparing predicted *Wolbachia* infection status with previously determined PCR-based infection status ( Verspoor & Haddrill, 2011). *Wolbachia* infection status was predicted from genome sequences for each strain following a modified protocol from Richardson *et al.* (2012). Briefly, strains were predicted as "infected" when breadth of mapped read coverage was greater than 90% of the *Wolbachia* genome and mean depth of coverage was greater than one. Here, we compute breadth of coverage directly from the bedtools genomecov (version v2.22.0) ( Quinlan & Hall, 2010) output rather than from a consensus sequence, as was done previously by Richardson *et al.* (2012). Predicted *Wolbachia* infection status matched experimentally determined infection status for 55/56 samples (98.2% concordance), indicating that strain or sample swaps are unlikely to have occurred during the generation of this dataset ( Dataset 1). The only exception observed was for line GA08 from the Georgia population, which the WGS data indicates is infected while PCR data indicates it is uninfected. This observation can be explained by either PCR amplification failure for the GA08 stock in Verspoor & Haddrill (2011) or infection of the GA08 stock after data collection for Verspoor & Haddrill (2011). Further analysis of the *Wolbachia* infection status of this stock is warranted prior to use.

## Data availability

Raw sequence data for the 56 samples reported here can be found in the European Nucleotide Archive ( http://www.ebi.ac.uk/ena) under accession ERP009059. Isofemale lines can be obtained from the *Drosophila* Species Stock Center ( https://stockcenter.ucsd.edu) under accessions 14021-0231.139, 14021-0231.140, 14021-0231.141, 14021-0231.142, 14021-0231.143, 14021-0231.144, 14021-0231.145, 14021-0231.146, 14021-0231.147, 14021-0231.148, 14021-0231.149, 14021-0231.150, 14021-0231.151, 14021-0231.152, 14021-0231.153, 14021-0231.154, 14021-0231.155, 14021-0231.156, 14021-0231.157, 14021-0231.158, 14021-0231.183, 14021-0231.184, 14021-0231.185, 14021-0231.186, 14021-0231.187, 14021-0231.188, 14021-0231.189, 14021-0231.190, 14021-0231.191, 14021-0231.192, 14021-0231.193, 14021-0231.194, 14021-0231.195, 14021-0231.196, 14021-0231.197, 14021-0231.163, 14021-0231.164, 14021-0231.165, 14021-0231.166, 14021-0231.167, 14021-0231.168, 14021-0231.170, 14021-0231.172, 14021-0231.174, 14021-0231.176, 14021-0231.177, 14021-0231.178, 14021-0231.180, 14021-0231.181 and 14021-0231.182..

Descriptive statistics for validation of each run can be found in Dataset 1, DOI: 10.5256/f1000research.6090.d42636 ( Bergman & Haddrill, 2014).
