# Supplementary material for: Strain-specific and pooled genome sequences for populations of Drosophila melanogaster from three continents
Source: F1000Res. 2015 Jan 29;4:31. [Version 1] doi: 10.12688/f1000research.6090.1 (PMC4331666; doi:10.12688/f1000research.6090.1)
Supplement: Peer review history

# Peer review: Referee response for version 1

**DOI:** 10.5256/f1000research.6521.r7634

**Published:** 2015-02-18

**Authors:** Ian Dworkin

**Affiliations:** 1 Department of Biology, McMaster University, Hamilton, ON, Canada

This article primarily summaries the generation of a large set of resequenced Drosophila strains from three populations (Ghana, France and the US). Sequencing was done both individually for each isofemale strain, as well as in sequenced pools for each of three populations. While the primary goal of this research appears to be to provide the community with these additional genomic resources, the researchers were also particularly interested in examining Wolbachia infection status in the strains. Given that all raw data has been made available, it is likely that will provide an important useful resource for genomic analyses.

A few minor comments:

Some comparison of mapping quality for the pooled sequences (as compared to the individual isofemale strains) would have been useful.

Some explanation as to why the number of individuals used for the three different sequencing pools differed would have also been helpful to understand the provenance of the data.

I have read this submission. I believe that I have an appropriate level of expertise to confirm that it is of an acceptable scientific standard.

# Peer review: Referee response for version 1

**DOI:** 10.5256/f1000research.6521.r7534

**Published:** 2015-02-03

**Authors:** John Pool

**Affiliations:** 1 Laboratory of Genetics, University of Wisconsin - Madison, Madison, WI, USA

The authors' data will add value to Drosophila population genomic resources.  I see no technical flaws in the manuscript.  If the authors see fit, they could a bit more context to the data.  For example, they could note that a mosaic of homozygous and heterozygous regions may be expected from the isofemale line genomes.  Optionally, they could also briefly put these three populations in historical context (i.e. that the species originated from sub-Saharan Africa but perhaps not western Africa specifically, that it expanded out of sub-Saharan Africa with a population bottleneck, and that North American populations are thought to have both European and African ancestry).  The France and Ghana samples sequenced here may prove useful for identifying population ancestry in North American and other admixed populations. 

Trivial edits:

Methods paragraph 1:

“each isofemale lines” (delete final “s”)

References - from title of Lack *et al.* 2015, delete second “genomes”.  Update precise author information.

I have read this submission. I believe that I have an appropriate level of expertise to confirm that it is of an acceptable scientific standard.
